# Variation in the Morphology of *Bacillus mycoides* Due to Applied Force and Substrate Structure

**DOI:** 10.1371/journal.pone.0081549

**Published:** 2013-12-06

**Authors:** James P. Stratford, Michael A. Woodley, Simon Park

**Affiliations:** 1 School of Biology, University of Nottingham, Nottingham, United Kingdom; 2 Vrije Universiteit Brussel, Center Leo Apostel for Interdisciplinary Studies, Brussels, Belgium; 3 Faculty of Health and Medical Sciences, University of Surrey, Guildford, United Kingdom; LAAS-CNRS, France

## Abstract

Response to mechanical force is a well characterised phenomenon in eukaryotic organisms, helping to organise multicellular structures. Mechanotactic responses have only rarely been observed in prokaryotic taxa. This work reports on a morphological change due to variations in applied force and surface structure by *Bacillus mycoides* Flügge. *B. mycoides* is a ubiquitous soil organism well known among microbiologists for its characteristic spreading colony morphology. An apparent mechanotactic response is elicited by physical deformation of the gel media on which *B.mycoides* is growing, including applied forces of compression or tension. Variations in the surface such as curvature produced by casting the agar gel in the presence of curved objects also elicited the change. The morphological change in *B.mycoides* colonies associated with the application of force manifests as a pattern of parallel rhizoid filaments perpendicular to compressing force and parallel to stretching force in the agar medium. The phenomenon is most clearly demonstrated by reversible changes in the orientation of *B. mycoides* filaments during time-lapse microscopy.

## Introduction

*Bacillus mycoides* Flügge is a member of the Firmicutes, low GC gram positive bacteria of ancient phylogeny. It is a spore forming rod which produces a characteristic spreading filamentary morphology when cultured on agar. *B. mycoides* is found in soils around the world however its niche has yet to be characterised [1], [2]. The closest relatives of *B. mycoides*–the *Bacillus cereus* subgenus- are pathogens of humans (*B. cereus, B. anthracis*) and other animals (*B. anthracis*) including insects (*B. thurigiensis*) [2]. *B. mycoides* has been known since 1886 when its spreading rhizoidal colony structure was first observed in soil cultures by Flügge [3]. *B. mycoides* is regarded as a saprophytic organism, which makes it ecologically distinct from its more pathogenic close relatives [4].

### Microbial Elasticotaxis

The response of microbes to force in their growth medium was first observed in *Myxococcus xanthus* [5]. *Myxobacteria* where found to be capable of aligning their cells perpendicular to compressive forces and parallel to extensive forces leading to colonies possessing a stretched appearance [5]. Stanier named this phenomenon in microbes “elasticotaxis”. Elasticotaxis in *Myxobacteria* has been observed to occur during motility, *Myxobacterial* cells have been observed to reorient the longitudinal axis of the cell when force is applied. This response has been experimentally associated with the A or “adventurous” motility system in this family of bacteria [6]. Mechanotaxis has not been commonly observed among other taxa of bacteria.

### Experimental Procedures

#### Strain

The strain of *B. mycoides* used for the experimental demonstrations is the direct descendent of that originally studied by Flugge (DSM 2048). This strain was used for the experiments presented here as its genetic identity had been confirmed by 16 s ribotyping (Gene Bank Accession Number: AB592538.1).

#### Culture media

Prior to growth on solid media *B.mycoides* was cultured overnight in 10 ml of nutrient broth in glass universals. For solid cultures of of *B. mycoides* Oxoid’s standard plate count agar (PCA) with a final agar concentration of 0.9% was used. The temperature used for culturing *B. mycoides* was 30°C. All media were purchased from Oxoid. All solid media were prepared in petri dishes which were inverted for 24 hrs in an incubator at 30°C to ensure a liquid-free but still hydrated surface. This was done before all cultures involving solid media.

#### Growth of B.mycoides under compressive force

For experiments investigating the response of *B.mycoides* to mechanical force an identical assay to that of Stanier [5] was used. More recent work by Fontes and Kaiser [6] has also employed the same methodology. The technique involves introducing a compressive strain in agar by placing an object between the side of a petri dish and the set agar in such a way as to distort the gel. Square petri dishes were filled with plate count agar, once the agar medium had set and cooled, force was applied to the medium by inserting 2 sterile microscope slides between the vertical edge of the plate and the edge of the agar. The cumulative thickness of the slides was 2 mm and produced in the agar a compressive strain of 0.022 (strain is a dimensionless number which represents fractional change in length under compressive force). Immediately following this procedure five microliter aliquots of overnight broth culture were inoculated onto the agar surface. Inoculation was carried out at 3 separate locations on each agar surface. Plates were then incubated for 24 hrs at 30°C. To improve photographic contrast pH neutral powder activated charcoal was added to the PCA medium. This was purely for enhanced image quality and did not affect the elastotactic response when compared to charcoal free PCA under the same conditions.

#### Well assay

To determine if *B. mycoides* morphology could change in response to nutrients, a wide range of soluble sugars and amino acids were deposited in 10 mm wells cut into standard PCA agar using a sterile cork borer. The assay consisted of one plate per substance with two wells and a single equidistant point between the two wells on to which 5 l of B. mycoides broth was inoculated. A 250 µl aliquot of 2% (by mass) solution of the test substance was added to one well on each plate with 250 µl of RO water added to the second well as a control. This process was carried out for three replicate plates for each substance. Plates were examined for any variations in appearance which could indicate morphological variation. The substances tested were the sugars: arabinose, galactose, glucose, sucrose and trehalose. The amino acids used were: Alanine, arginine, asparagine, aspartic acid, cysteine, glutamine, glycine, leucine, lysine, methionine, phenylalanine, tryptophan and valine.

#### Orientation of filaments on curved surfaces

For experiments investigating the effect of curvature on filament direction, molten agar in 9 cm circular plates was impressioned with the base of sterile 250 ml round bottomed flasks. The impressioning was carried out by leaving the flask in place until the agar had cooled and solidified. The flask was then carefully removed and the agar inspected to ensure a continuous surface. The absolute centre of each impression would often have a very small area of exposed petri dish not covered by agar due to displacement at the point of contact between the base of the petri dish and the flask. This was corrected by the addition of 25 µl of molten PCA to this central point prior to the drying of the plate. Impressioned PCA plates were inoculated with 5 microliters of *B. mycoides* broth and incubated for 24 hours at 30°C.

#### Morphological change with simultaneous compression and tension

To determine if the response was coordinated at a colony level or at the level of individual filaments, both compressive and stretching strain were introduced simultaneously in a single section of agar and a single colony allowed to over-grow both regions. Another of the culture the methods of Stanier [5] was used to produce the simultaneous tension and compression: a rectangular section of agar was cut out and transferred aseptically to sterile petri dish. The rectangular section was placed over a section of a 5 mm diameter sterile plastic rod resulting in an upward curvature in the middle of the agar section. This produces tension in the top side of the upward inflected region while producing compression at the inward inflected angles either side. Following the application of the distorting rod, the centre of the upwardly curved region was inoculated with 5 µl of broth such that the colony would grow over the stretched region and then transition to growth on the compressed region. Following 24 hrs of incubation at 30°C the rod was removed from under the agar slice and images taken of the colony morphology.

#### Time-lapse imagery of changes in orientation

*B. mycoides* was inoculated on to charcoal free PCA in square petri plates. Compression was applied by insertion of two microscope slides between the agar and the side wall of the petri dish. This assembly was incubated at 30°C for 12 hrs then transferred to a heated stage for *in-situ* incubation at 30°C during time lapse. For the duration of time-lapse the direction in which the force was applied was shifted by 90° (at *t* = 0). Initial force application during culture was used to ensure a uniform mono-directional alignment of filaments before the direction of the force was changed. Force was applied using two microscope slides inserted between the edge of the petri dish and the agar producing a compressive strain of 0.022. Time-lapse imagery of mechanotactic responses in *B. mycoides* filaments was accomplished using a leitz dialux phase contrast microscope at 100× magnification (10× objective). Changes in the direction of the force were accomplished by removing the microscope slides from the end of the square plate and replacing them on the adjacent side. This produced a force 90° rotation from the previous direction. Images were collected using a Brunel microscopes digital CCD camera coupled to a laptop computer for image capture. Images were collected automatically at 5 minute intervals.

#### Bead tracking

To demonstrate that mechanotaxis causes *B.mycoides* to interact with objects in its surroundings, sterile 5 mm glass beads were placed on the surface of plate count agar in petri dishes. The plates were then inoculated with 5 µl of 24 hr broth culture of *B. mycoides*. Cultures were incubated for 24 hours at 30°C and examined for variations in morphology.

#### Photography

Macroscopic images of colonies were taken using a Cannon EOS 40D equipped with a Canon EF100 mm macro lens.

## Results

### Growth Under Compressive Force

**Figure 1:**
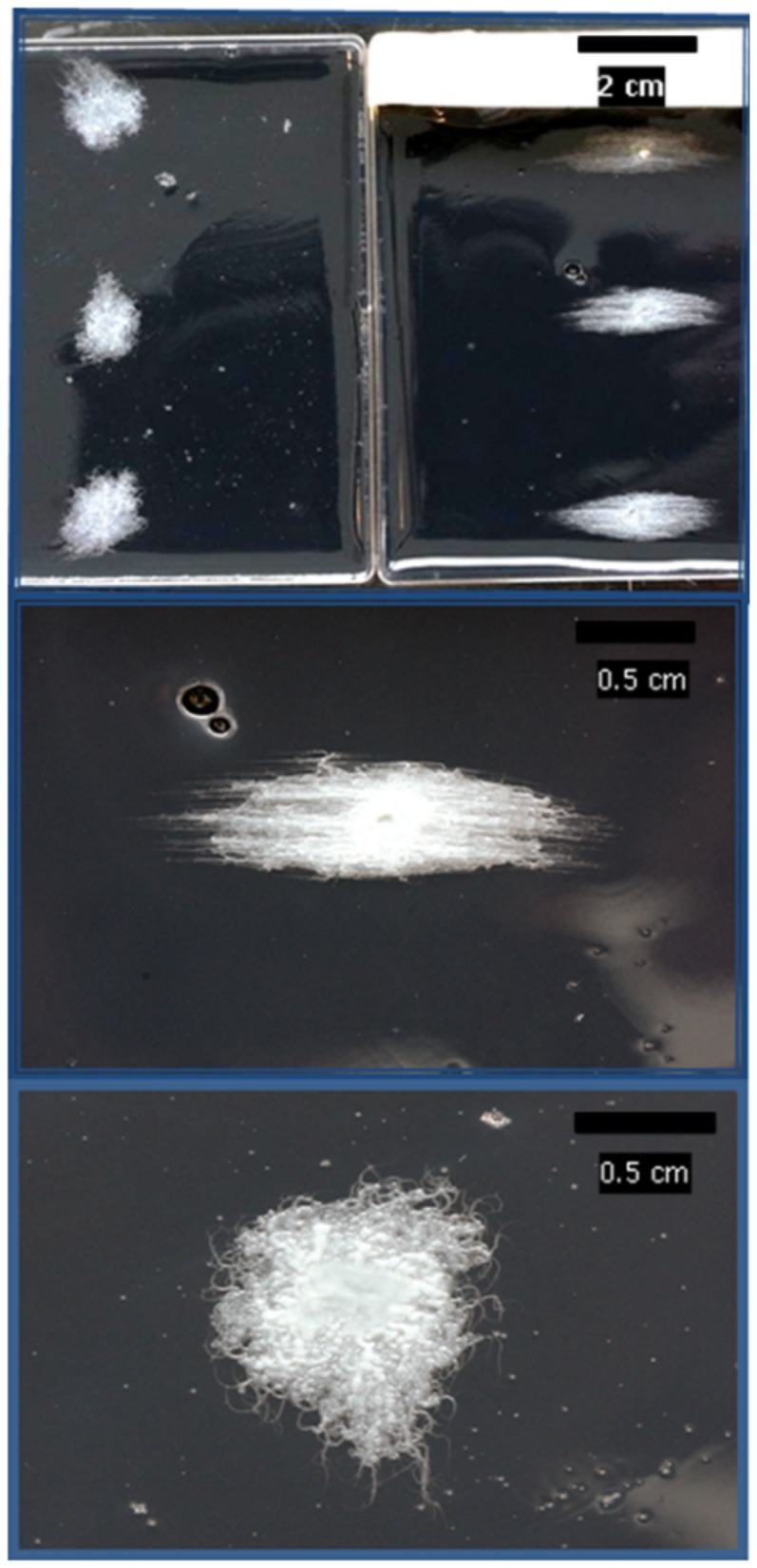
Comparison of the colony morphology of B. mycoides. Control (top left) and under compression (top right). The white strip on top right is a tape restraining the slides in the vertical position. Centre and bottom: close-up of colony morphology under conditions of compression and control respectively.

The methodology employed in producing figure 1 is identical to that originally employed by Stanier [5] and more recently published by Fontes and Kaiser [6]. Figure 1 demonstrates the response to force of *B. mycoides* DSM 2048. *B. mycoides* was capable of responding to the direction of compression force in the solid media on which it was grown. This response is visible in the three replicate colonies on the compressed plate and absent in the three colonies on the control plate. Filaments which usually form a self-similar chiral morphology become straightened and parallel. The direction of growth observed when force was applied was perpendicular to the direction of the compression by the microscope slides.

The pattern of response visible in figure 1 was common to all colonies grown on PCA without the charcoal or on nutrient agar in place of PCA. Only the colonies grown on the charcoal containing PCA medium are shown due to the high contrast required to see details in these small structures. This method has the advantage of allowing sub-millimetre resolution making individual filaments of B.mycoides visible to normal macro photography.

### Orientation of Filaments on Curved Surfaces

**Figure 2:**
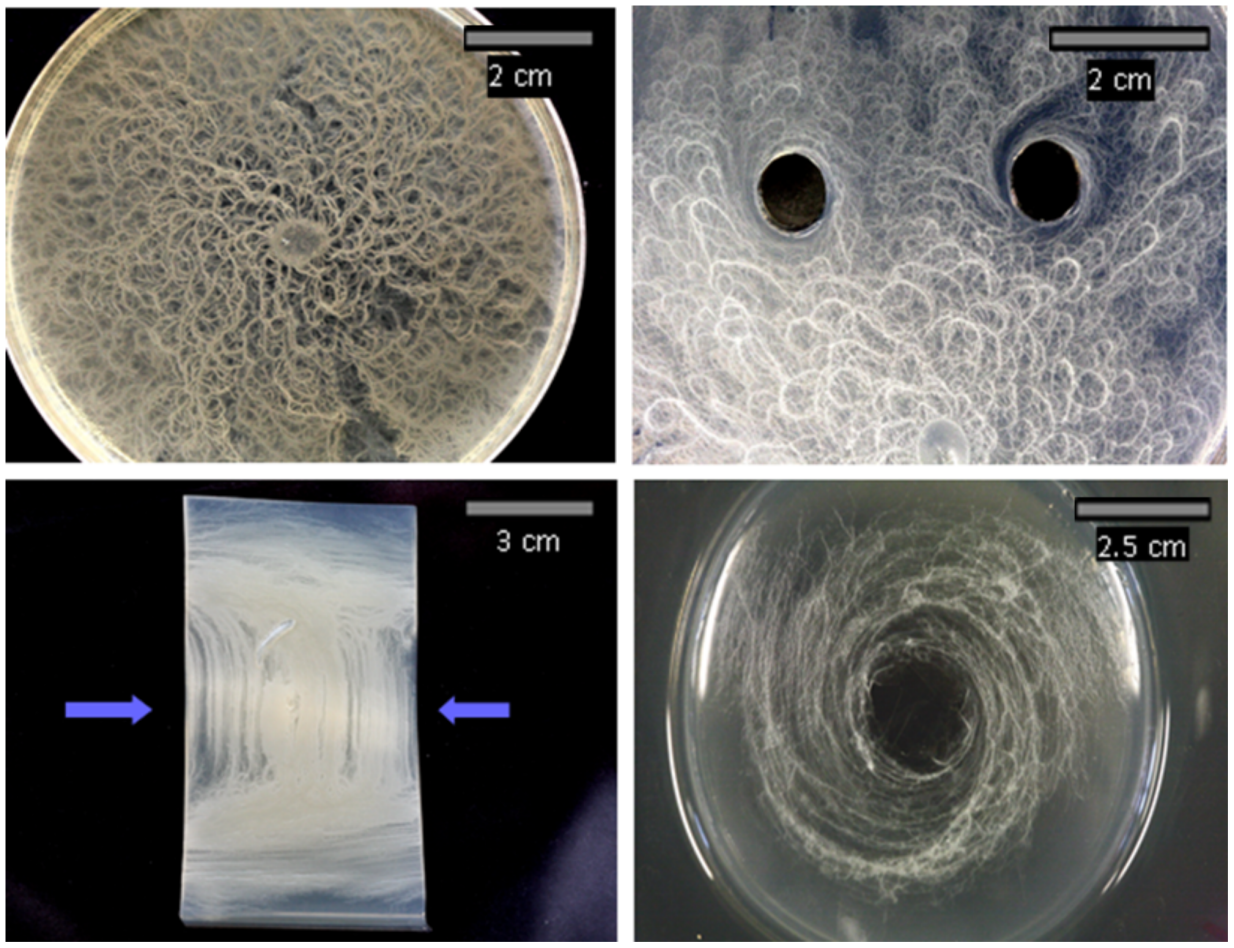
The effects of altered substrate structure on the colony morphology of B. mycoides. The normal chiral morphology of B. mycoides when grown from a single central point of inoculation on plate count agar (Top left). The response of a single propagating colony of B. mycoides to wells cut in the agar (Top right). The response of a single propagating colony of B. mycoides to forces of tension and compression introduced into plate count agar (Below left). The blue arrows indicate the position of a 5 mm glass rod underneath the agar slab during culture (removed for imaging). The response of a single propagating colony of B. mycoides to a continuous curved gradient in the agar gel introduced by casting the gel around the base of a round- bottomed flask (Below right).

When curvature was introduced into the substrate, the filaments responded by growing in a spiral pattern oriented around the centre of the curvature. This pattern resembled that observed around the test wells. The pattern can be seen in figure 2. It is not known whether the response to curvature is mediated by force as no force is intentionally applied after the casting of the agar. It is possible that residual forces are present in the agar gel after it has set which could influence the orientation of the filaments.

### Morphological Changes Under Simultaneous Compression and Tension

The filaments of *B. mycoides* responded to force by growing in a direction parallel to extension strain and perpendicular to compression strain. This produced the pattern visible in figure 2 where the filaments are vertically aligned in the longitudinal centre of the agar but become horizontally aligned as they enter the region of compression above and below the centre. This demonstrates that single propagating colonies can simultaneously exhibit different filament orientations as a result of varying orientation in force. This also suggests filament orientation and morphology are not coordinated at the colony level.

### Time-lapse Imagery of Changes in Orientation

**Figure 3:**
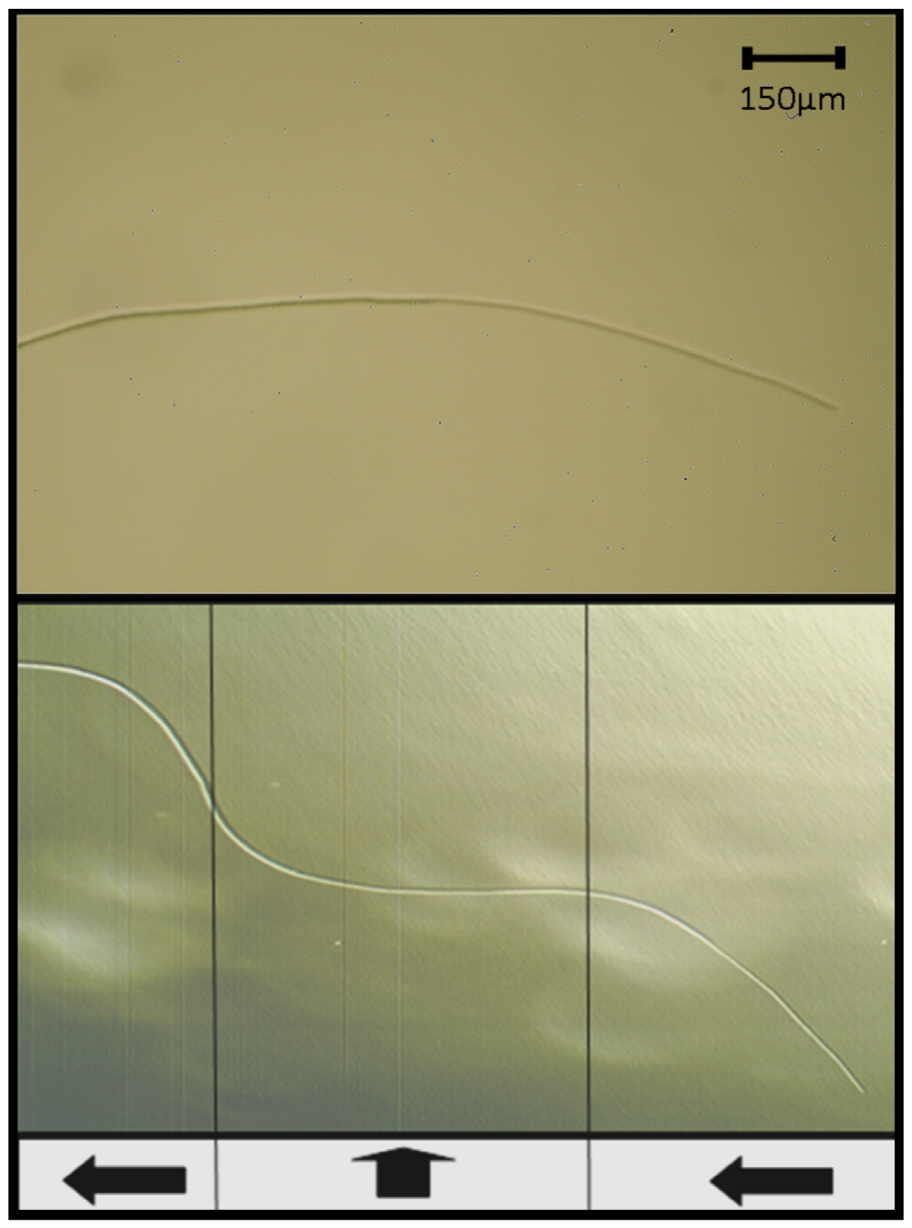
Demonstration of a single filament responding to forces applied in different directions during filament propagation. Above: A control filament grown without the influence of applied force. Below: Filament growth under compression. Vertical lines represent the transition of force direction and are separated by 45 min time spans. Black arrows indicate the direction of compression in the medium.

**Figure 4:**
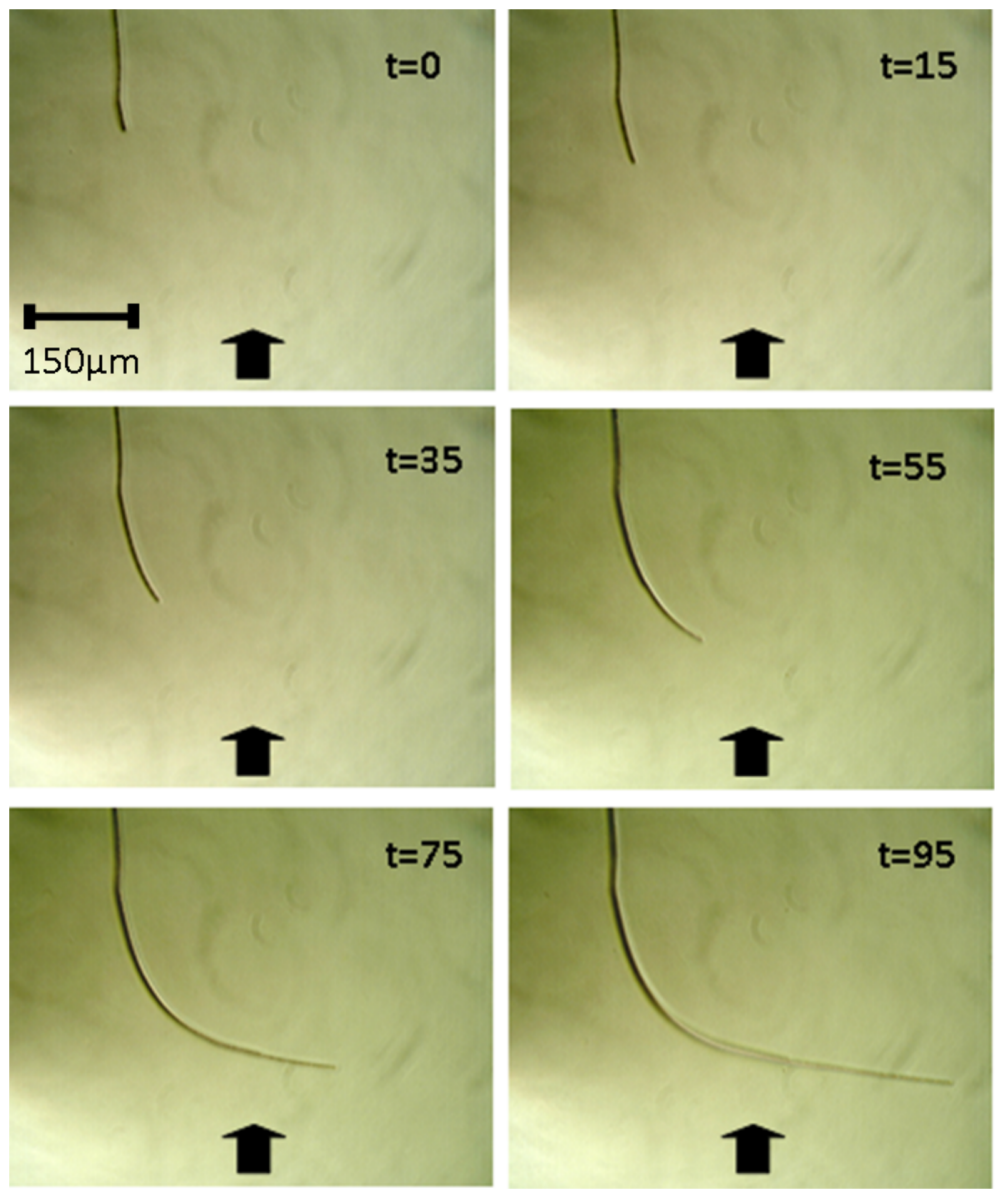
Time-lapse images of a single *B. mycoides* filament responding to the application of compressive force. Time is indicated in minutes.

Application of force can allow the steering of a filament such that more than one curve is present in the final structure. The lower image in figure 3 shows the effect of twice changing the direction of force in 45 minute intervals alongside a control filament with no force applied (above). The filament produces a stepped structure with 3 inflections reflecting the changing forces during the establishment of the filament. The resulting structure would not be expected to occur naturally and indicates that the leading edge of the filament is being influenced by direction of force in the medium. Figure 3 also shows that individual filaments which have previously changed orientation as a result of force in one direction can subsequently reorient as a result of a new direction of force. Time-lapse measurements demonstrate that the mechanotactic response is progressive and occurs due to the gradual realignment of the colonies leading edge filaments (see figure 4). This realignment process is first noticeable at 35 mins and is very clear by 55 mins. By 95 mins the filament has turned 80° after which the direction of growth appears to be set. It can be seen that the filament does not move with respect to the agar other than the extension at the leading edge. Curvature only occurs due to a change of direction at the apex of the filament. Filaments can cover a distance of half a millimetre (about one hundred cell lengths) in one hour (see fig. 3 and 4). This is far faster than could be achieved by a single cell dividing at the apex as this would require 50 divisions in one hour. This suggests that the leading region of the filament shifts over the surface as constituent cells divide in unison. Such a process is distinct from other modes of filamentary growth such as that observed in fungi and actinomycetes which spread by extension of the filamentary apex [7]. It is tempting to speculate that the gliding of cells over the agar surface makes the filaments course sensitive to small surface imperfections, grooves or molecular realignment produced by force in the substrate.

### Bead Tracking by Filaments of B.mycoides

**Figure 5:**
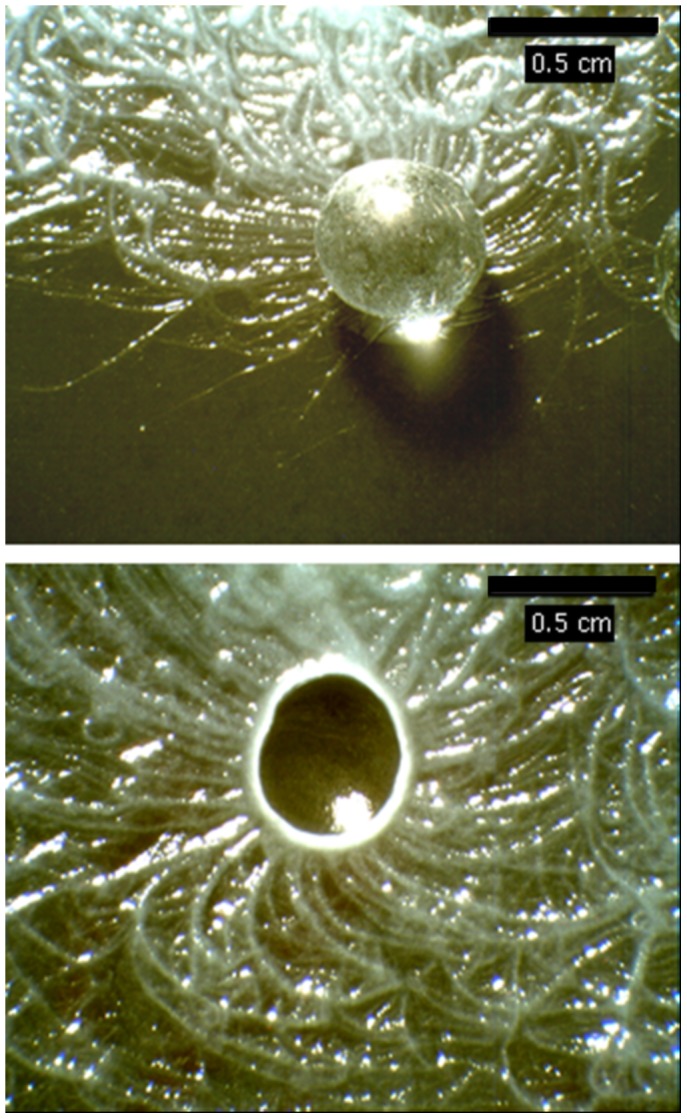
Variation in B. mycoides colony morphology due to the presence of 5 mm glass beads during incubation on PCA: with bead still in place (top); with bead removed (below).

Filaments of *B. mycoides* respond to the presence of glass beads by curving towards them. As can be seen in figure 5 this response begins at a distance of around 0.5 cm from the edge of the bead. The result is a pattern of filaments surrounding the bead and curving inwards to the position of the bead on the agar surface. This radial pattern of filaments is most clearly visible when the bead is removed.

## Discussion

It has been shown that *B.mycoides* is capable of responding to force and structural deformation in the agar medium on which it is cultured. The discovery of elasticotaxis in *B. mycoides* suggests the phenomenon is more widely distributed with regard to microbial taxa than previously thought. The mechanisms by which *B.mycoides* can respond to mechanical force are not known but some clues as to possible mechanisms are present in the literature on Myxobacterial motility. It is important to note that in the case of *B. mycoides* no mechanism for cell motility has been observed. Morphological responses occur due to the curvature of the path taken by these leading edge filaments. A number of hypotheses for the mechanism can be proposed, either *B. mycoides* cell change their curvature by variation in cell wall assembly or filaments are flexible and change course due to a molecular motor in the cell envelope. Insights into the possible mechanisms for this second option are available from the literature on Myxobacterial motility. Myxobacterial adventurous (A) motility has been shown to involve helically distributed molecular motors in the cell envelope of *Myxococcus xanthus* [8], [9], [10]. Myxobacterial adventurous motility is also exclusively associated with elasticotaxis in *M. xanthus* [6]. Alternatively the intimate contact B.mycoides filaments have with their substrate causes their direction to vary in line with (as yet unobserved) microscopic surface imperfections or molecular re-arrangement of gel polymer molecules occurring due to the applied force. This last explanation would also answer the conundrum of why the bacterium can respond in a similar fashion to curvature in the medium not produced by force.

The organisation and collective motion of cells are important processes in the context of understanding tumour metastasis and tissue morphogenesis [11]. Mechanotaxis is also an important process in the organisation of eukaryotic tissues. Uncovering the mechanisms/s of mechanotaxis may lead to a deeper understanding of the forces involved in the propagation of masses of cells as well as the processes determining their structural patterns. Finally a novel potential application of our findings with *B. mycoides* may be in the field of engineering, where the phenomenon may be useful for mapping strain in hydrogels. With further understanding, the process of filament guidance could also be used as a novel technique for producing complex microscopic structures. This last point would be especially relevant if physical processes rather than molecular mechanisms are responsible for the morphological changes observed as the underlying mechanism could more easily be applied to abiotic systems.

## References

[1] Buyer JS (1995) A soil and rhizosphere microorganism isolation and enumeration medium that inhibits *Bacillus mycoides* . Appl Environ Microbiol 61: 1839–1842. 16535025 10.1128/aem.61.5.1839-1842.1995 PMC1388443

[2] Ko KS, Kim JW, Kim JM, Kim W, Chung IJ, et al (2004) Population structure of the *Bacillus cereus* group as determined by sequence analysis of six housekeeping genes and the plcR gene. Infect Immun 72: 5253–5261. 15322020 10.1128/IAI.72.9.5253-5261.2004 PMC517475

[3] Flügge C (1886) Die mikroorganismen. Leipzig. FCW Vogel.

[4] Nakamura L, Jackson M (1995) Clarification of the taxonomy of *Bacillus mycoides* . International journal of systematic bacteriology 45(1): 4–7.

[5] Stanier RY (1942) A note on elasticotaxis in *Myxobacteria* . J Bacteriol 44: 405–412. 16560578 10.1128/jb.44.4.405-412.1942 PMC373690

[6] Fontes M, Kaiser D (1999) *Myxococcus* cells respond to elastic forces in their substrate. Proc Natl Acad Sci USA 96: 8052–8057. 10393946 10.1073/pnas.96.14.8052 PMC22186

[7] Goriely A, Tabor M (2008) Mathematical modelling of hyphal tip growth. Fungal Biology Reviews 22(2): 77–83.

[8] Nan B, Chen J, Neu JC, Berry RM, Oster G, et al (2011) *Myxobacteria* gliding motility requires cytoskeleton rotation powered by proton motive force. Proc Natl Acad Sci USA 108: 2498–2503. 21248229 10.1073/pnas.1018556108 PMC3038734

[9] Pate JL, Chang LYE (1979) Evidence that gliding motility in prokaryotic cells is driven by rotary assemblies in the cell envelopes. Curr Microbiol 2: 59–64.

[10] Spormann AM (1999) Gliding motility in bacteria: insights from studies of *Myxococcus xanthus* gliding motility. Microbiol Mol Biol Rev 63: 621–641. 10477310 10.1128/mmbr.63.3.621-641.1999 PMC103748

[11] Trepat X, Wasserman MR, Angelini TE, Millet E, Weitz D, et al (2009) Physical forces during collective cell migration. Nature Physics 5(6): 426–430.

